# The relationship between teacher care behavior and English learning performance: a serial multiple mediation model

**DOI:** 10.3389/fpsyg.2025.1617592

**Published:** 2025-09-10

**Authors:** Dongmei Wang, Qiusheng Wu, Cong Lei

**Affiliations:** ^1^School of Foreign Languages, Hubei University of Economics, Wuhan, China; ^2^Department of Foreign Languages, Wannan Medical College, Wuhu, China; ^3^College of Overseas Education, Sanming University, Sanming, China

**Keywords:** teacher care behavior, learning motivation, learning engagement, self-efficacy, English learning performance

## Abstract

This study explores the link between Teacher Care Behavior (TCB) and English Learning Performance (ELP), examining a serial multiple mediation of English Learning Motivation (ELM), English learning Engagement (ELE), English Self-efficacy (ESE), and English learning Strategies (ELS). A questionnaire survey assessed these factors, with ELP based on CET-4 (College English test-band 4) scores. The serial multiple mediation model revealed TCB positively predicts ELP through two motivational-cognitive chains based on Self-Determination Theory (SDT) and Social Cognitive Theory (SCT). The SDT-driven motivational pathway is TCB → ELM→ELE → ELP and SCT-driven cognitive-efficacy pathway is TCB → ESE → ELS → ELP. These findings offer new insights into TCB and student psychological mechanisms in foreign language (FL) learning, providing a theoretical and practical basis for optimizing the learning environment and enhancing outcomes.

## 1 Introduction

In many countries, like China, English is regarded as the most important FL and highly valued. However, for many students, English learning remains a significant challenge, becoming a bottleneck to their academic and career development (Wang, 2023). Therefore, exploring the factors influencing English learning, particularly the interaction between the environment, such as teachers and students’ psychological mechanisms, has become a hot topic in the field of FL education (Ma et al., 2017, 2022; Sun, 2021; Wang, 2023).

Following Lei (2014), Teacher Care Behavior (TCB) is defined as a multidimensional construct encompassing: conscientiousness, such as diligent task completion, instant homework feedback, fairness in rewards and punishment, strictness with students’ learning etc., support, manifested in academic/emotional investment such as individualized feedback, taking time to know, concern and interact with students etc., and inclusiveness, such as accommodating student needs, accepting students’ weakness, encouragement of class questioning and answering, etc. Crucially, TCB differs from general teacher support by emphasizing proactive emotional attunement rather than reactive aid (O’Connor, 2008). Despite established evidence linking TCB to academic outcomes (Lei et al., 2015), three critical theoretical and contextual gaps persist in EFL literature: (1) Mechanistic gap: While prior studies confirm direct TCB-outcome links, such as Wang’s (2023) anxiety reduction and Schunk and DiBenedetto’s (2021) engagement, they neglect sequential mediation pathways in FL context; (2) Theoretical gap: Existing work rarely integrates SDT (motivation-driven) and SCT(efficacy-driven) pathways to explain TCB’s holistic impact; (3) Contextual gap: Existing research disproportionately examines middle school learners like Ma et al. (2017) or non-FL subjects, like Lei et al. (2015), overlooking university EFL learners where language proficiency critically impacts academic/career trajectories.

**Figure 1:**
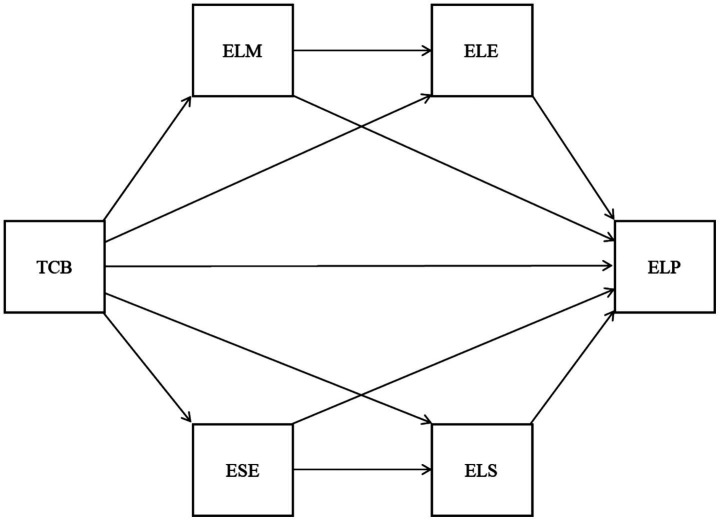
The hypothesized serial multiple mediation model between TCB and ELP through two sequential mediators.

This study addresses these gaps by proposing a dual-pathway serial mediation model (Figure 1) grounded in two established theories: Self-Determination Theory (Deci and Ryan, 2000), which explains how TCB satisfies students’ psychological needs to enhance motivation (ELM) and engagement (ELE) and Social Cognitive Theory (Bandura, 1986), which elucidates the role of TCB in building self-efficacy (ESE) and facilitating strategy use (ELS), to enhance ELP among Chinese university learners—a novel contribution bridging theoretical fragmentation in EFL scholarship, which holds great significance for FL teaching and learning.

## 2 Literature

### 2.1 Theoretical framework: integrating SDT and SCT

Self-Determination Theory (Deci and Ryan, 2000) posits that human motivation and behavior are driven by the fulfillment of three innate psychological needs: autonomy (volitional control), competence (mastery of tasks), and relatedness (social connectedness). In educational contexts, teacher behaviors that satisfy these needs—particularly relatedness through care and emotional support-enhance intrinsic motivation, foster engagement, and ultimately improve academic performance (Niemiec and Ryan, 2009; Ryan and Deci, 2017). TCB operationalizes SDT’s relatedness support by providing individualized attention (e.g., constructive feedback) to fulfill autonomy needs (Reeve, 2016), creating a low-anxiety environment to bolster competence perceptions (Wang, 2023) and building trusting teacher-student relationships to strengthen relatedness (Lei et al., 2015). SDT has been widely applied to explain language learning motivation, where teacher emotional support predicts English Learning Motivation through need satisfaction (Ma et al., 2022); autonomous motivation mediates the relationship between teacher support and English Learning Engagement (Oga-Baldwin, 2019); SDT-based interventions (e.g., student-centered tasks) improve EFL performance (Ryan and Deci, 2017; Pishghadam et al., 2021).

Social Cognitive Theory (Bandura, 1986) posits that human behavior is shaped through triadic reciprocal causation, where personal factors (e.g., cognition, affect), environmental influences (e.g., teacher behaviors), and behavioral patterns (e.g., learning strategies) interact dynamically. Central to SCT is the construct of self-efficacy—an individual’s belief in their capability to execute actions required to achieve specific goals (Bandura, 1977). In educational settings, teachers play a pivotal role in fostering students’ self-efficacy through mastery experiences (e.g., scaffolded tasks that ensure success), vicarious learning (e.g., modeling effective strategies), verbal persuasion (e.g., constructive feedback), and emotional arousal regulation (e.g., reducing anxiety) (Bandura, 1977; Schunk and DiBenedetto, 2021). TCB operationalizes SCT’s environmental influence by providing mastery experiences through tailored support (e.g., differentiated instruction) to enhance English self-efficacy; modeling of strategies from teachers (e.g., metacognitive planning) enhances English learning strategies adoption, particularly under self-efficacy conditions; creating an emotionally safe classroom to reduce efficacy-undermining anxiety (Wang, 2023). SCT has been extensively applied to explain EFL learning processes: Teacher support directly predicts self-efficacy, which in turn influences language achievement (Ma et al., 2017); High self-efficacy students employ more cognitive and metacognitive strategies (Teng and Yang, 2023); SCT-based interventions, such as strategy training significantly improve EFL performance (Ghonsooly and Elahi, 2010; Ma et al., 2017).

### 2.2 Learning engagement and performance

Learning engagement refers to the degree to which students participate in the learning process, including behavioral, emotional and cognitive engagement (Skinner and Belmont, 1993). Learning engagement is one of the most important predictors of language learning success (Mercer and Dörnyei, 2020), and its importance has received attention and research from numerous scholars (Philp and Duchesne, 2016; Sulis, 2022; Tsang and Dewaele, 2023; Wang and Xue, 2024). There are many factors affecting learning engagement, such as personal attitude, teacher’s teaching and school support et al., among which teachers are proven an important factor influencing students’ learning engagement (Oga-Baldwin, 2019; Wang, 2023). A good teacher-student relationship can create a sense of joy and promote their learning engagement (Cui and Hu, 2022).

English Learning Engagement (ELE) denotes learners’ multidimensional involvement in EFL activities, including behavioral (effort persistence), emotional (positive attitudes), and cognitive (self-regulation) components (Fredricks et al., 2004). Research has shown the positive impact of FL learning engagement on learners’ language acquisition (Guo, 2018). English learning engagement can positively predict English learning outcomes (Wang and Xue, 2024). Through a survey of 320 college students from three universities in a province of China, Guo (2018) found that there is a direct positive correlation between English learning engagement and academic performance.

### 2.3 Learning motivation, engagement, and performance

Motivation is driving force for an individual to engage in a certain behavior, including intrinsic motivation and extrinsic motivation (Atkinson et al., 1953). Intrinsic motivation stems from interest in the activity, representing the highest self-determination. Extrinsic motivation, triggered externally can become intrinsic through internalization, boosting activity engagement. Self-determination theory (SDT) posits motivation arises from satisfying autonomy, competence and relatedness (Deci and Ryan, 2000). By meeting these needs, individuals enhance internal motivation, promote external motivation internalization, driving self-determination and self-actualization (He and Zhou, 2022). Motivation satisfaction is key for behavioral engagement and self-actualization.

English Learning Motivation (ELM) refers to learners’ driving force to engage in English acquisition, encompassing intrinsic (e.g., interest in language) and extrinsic (e.g., academic requirements) dimensions (Deci and Ryan, 2000). Language learning researchers pay great attention to learners’ motivation (Gardner and Lambert, 1972), which can drive students to engage in learning activities, and has a significant impact on academic performance (Wang and Wang, 2025). There is a close relationship between learning motivation, engagement and performance. Studies have shown that the higher a student’s level of learning motivation, the more effort they engage in their studies (Hidi and Harackiewicz, 2010). FL learning motivation positively predict learning engagement (He and Zhou, 2022), and the higher the FL learning motivation, the greater the learning engagement (Yang and Dai, 2021). Motivation also has a significant impact on academic performance (Ma et al., 2022), which is related to the quality of FL learning performance (Liu and Dong, 2023). This is because students with strong motivation are more inclined to actively learn and exhibit higher levels of learning engagement, including active participation in behavior, positive emotional experiences, and deep cognitive processing, in order to fully absorb and master knowledge. Through this continuous effort and investment, it ultimately transformed into excellent academic performance.

### 2.4 Learning strategies and performance

Learning strategies play a crucial role in language learning, as they are specific actions taken by learners to acquire, understand, store, and retrieve information more effectively (Weinstein and Mayer, 1983), and also one of the key factors determining the success or failure of language learning (O’Malley and Chamot, 1990). English Learning Strategies (ELS) are deliberate techniques (e.g., metacognitive planning, memory aids) employed to optimize language acquisition (Oxford, 1990). Oxford divided language learning strategies into six categories: memory strategies, cognitive strategies, compensation strategies, metacognitive strategies, affective strategies, and communicative strategies. These strategies cover various aspects of language learning, from basic memory skills to complex emotional management and communicative interactions, and comprehensively affect learners’ language learning process.

Studies have shown learning strategies have a significant impact on academic performance (Yang et al., 2021; Teng and Yang, 2023). Learning strategies not only positively predict academic performance (Qin et al., 2024), but also effectively alleviate the negative impact of learning disabilities on academic performance (Bressane et al., 2024). This means students who effectively apply learning strategies are more likely to succeed academically, especially when facing learning challenges and difficulties. In the context of language learning, the importance of learning strategies is particularly prominent, as they have a significant impact on learning engagement, motivation and performance (Liu and Chen, 2024). This emphasizes the important role of learning strategies in language learning, suggesting educators and learners should pay attention to the cultivation and application of learning strategies to improve the efficiency and effectiveness of language learning.

### 2.5 Self efficacy, learning strategies and performance

Self-efficacy is an individual’s estimation and judgment of their ability to complete a specific task (Bandura, 1977), which is an important motivational variable affecting learning (Woodrow, 2011). Students with higher self-efficacy are more likely to believe they are performing better and therefore invest more energy and time in learning process. Research has shown a significantly positive correlation between self-efficacy and learning strategies (Ma et al., 2017; Teng and Yang, 2023). Students with stronger self-efficacy are more likely to actively use various learning strategies (Cui and Hu, 2022).

English self-efficacy (ESE) refers to students’ perception of whether they can make subjective judgments on successfully completing English language learning (Zhang and Yuan, 2004). There is a significant correlation between ESE and learning performance (Zhang et al., 2023; Ding, 2024). Self-efficacy not only directly affects learning performance, but also influences it through other mediating variables such as learning strategies, motivation and anxiety (Ma et al., 2017; Teng and Yang, 2023). Students with higher self-efficacy are able to experience more pleasant and positive emotions (Cui and Hu, 2022) and have lower levels of learning anxiety. Meanwhile, individuals with high self-efficacy tend to use more flexible and diverse learning strategies and work harder to complete learning tasks, while those with low self-efficacy often lack the ability to monitor and regulate their own learning behavior (Teng and Yang, 2023), and tend to perceive learning tasks as difficult (Schunk and Pajares, 2010), leading to burnout and ultimately giving up on completing tasks (Cui and Hu, 2022).

### 2.6 TCB, learning motivation, learning engagement, self-efficacy, learning strategies and learning performance

TCB involves teachers’ diligent task completion, investment in student development, and accommodation of student behavior to foster a positive teacher-student relationship (Lei, 2014; O’Connor, 2008). Ma et al. (2022) argue that the teacher-student relationship has a significant impact on students’ academic achievement. A supportive teacher-student relationship is beneficial for students’ academic development, while an unfavorable teacher-student relationship is detrimental to students’ academic development. TCB is conducive to creating a good teacher-student relationship. Research has shown a good teacher-student relationship has a positive impact on students’ learning motivation and improves the academic performance of struggling students (Ma et al., 2022). TCB can stimulate effective learning behavior, promote active participation (Wang and Shui, 2020), improve academic performance (Lei et al., 2015) and alleviate learning stress (Jiang et al., 2023).

Research has shown that teachers are one of the important factors affecting students’ engagement in FL learning (Mochklas et al., 2023). A good teacher-student relationship can reduce negative emotional interference, lower anxious behavior (Wang, 2023), trigger positive teacher-student interaction (Pishghadam et al., 2021), promote effective learning behavior and engagement (Sun, 2021), and improve the academic performance of EFL learners (Ma et al., 2022). At the same time, there is a close relationship between teachers and student’ s self-efficacy. Teachers’ teaching attitudes, styles, self-confidence, and infectiousness will have a significant impact on students’ learning confidence (Tian, 2013). Through a study of 11,036 eighth grade middle school students in China, Ma et al. (2017) found that the teacher-student relationship positively predicted FL performance, with self-efficacy and learning strategies playing an important mediating role in it.

From the above literature, we can see that prior work is fragmented: Ma et al. (2017) linked teacher-student relationships to self-efficacy/strategies but omitted motivation; Wang (2023) tied TCB to anxiety reduction without testing performance pathways. Our model integrates these fragments through SDT/SCT, proposing TCB triggers motivational-cognitive chains (Figure 1) unexplored in EFL literature.

### 2.7 Research hypothesis and model

Building on the integrated SDT/SCT framework (Figure 1), we hypothesize that:

H1: TCB, ELM, ELE, ESE, ELS, and ELP positively correlate with each other.
H2: ELM mediates the relationship between TCB and ELP.
H3: ELE mediates the relationship between TCB and ELP.
H4: ESE mediates the relationship between TCB and ELP.
H5: ELS mediates the relationship between TCB and ELP.
H6: ELM followed by ELE serially mediate the relationship between TCB and ELP.
H7: ESE followed by ELS serially mediate the relationship between TCB and ELP.

The model illustrates how TCB’s effects cascade through motivational (ELM→ELE) and cognitive-efficacy (ESE → ELS) pathways to enhance ELP.

## 3 Methods

### 3.1 Participants

Data were collected from three public universities in Central and Southeast China, which have large student populations and diverse disciplines and where English is a 1 ~ 2-year compulsory course and 4-year optional course. These institutions emphasize CET-4 as a graduation requirement, with curricula focused on standardized test preparation. Average CET-4 pass-rates at these universities range from 55 ~ 85% (national average for the first time: ~40%), reflecting moderate-to-high EFL teaching resources. The participants answered the questionnaire and provided their CET-4 scores. A total of 568 students participated in this survey. Finally, there were 503 students (excluding students with incomplete information and online answering time less than 180 s) with an average age (M ± SD) of 20.053 ± 1.509. The demographic information is listed in Table 1.

**Table 1:** Participant demographics (*N* = 503).

Variable	Category	*n* (%)
Gender	Female	346 (68.78%)
	Male	157 (31.22%)
Major	Humanities	257 (51.09%)
	Science/Engineering	246 (48.91%)
Grade	Freshmen	103 (20.47%)
	Sophomores	163 (32.41%)
	Juniors	166 (33.00%)
	Seniors	71 (14.12%)
Age (M ± SD)		20.053 ± 1.509

### 3.2 Measures

The questionnaire consists of five scales, namely the TCB, ELM, ELE, ESE, and ELS Scales. Their reported CET-4 scores were used to measure students’ ELP.

#### 3.2.1 TCB scale

The TCB scale developed by Lei (2014) consists of 18 items and three dimensions: conscientiousness, support, and inclusiveness. Students were asked to report their teacher’s behavior during their college English learning (e.g., The teacher takes time to know me). The scores were rated using a Likert 5-point scoring system, from “1” strongly disagree to “5” strongly agree, the higher the score, the higher TCB. The scale underwent rigorous psychometric validation, including exploratory and confirmatory factor analyses. All indices met established thresholds for good model fit: Reliability: Cronbach’s *α* = 0.937, Sampling adequacy: KMO = 0.935, Sphericity: Bartlett’s test = 7080.856 (*p* < 0.05). Structural validity: *χ^2^/df* = 2.417, GFI = 0.908, CFI = 0.926, TLI = 0.910, IFI = 0.926, RMSEA = 0.052.

#### 3.2.2 ELM scale

The ELM Scale developed by Lv and Yang (2013) includes six aspects: intrinsic interest, external requirements, cultural exchange, social responsibility, auxiliary tools, and personal development, totally 25 options. Students were required to answer questions about why they learn English during their college years (e.g., My enthusiasm in learning English largely depends on whether I like the English teacher or not). This scale adopts a Likert 5-point scoring system, ranging from “1” strongly disagree to “5” strongly agree, with higher scores indicating higher student motivation. The scale underwent rigorous psychometric validation, including exploratory and confirmatory factor analyses. All indices met established thresholds for good model fit:

Reliability: Cronbach’s *α* = 0.924, Sampling adequacy: KMO = 0.903, Sphericity: Bartlett’s test = 18485.423 (*p* < 0.05). Structural validity: *χ^2^/df* = 2.891, GFI = 0.925, CFI = 0.957, TLI = 0.942, IFI = 0.957, RMSEA = 0.043.

#### 3.2.3 ELE scale

The ELE scale developed by Ding (2017) includes three aspects: behavioral engagement, emotional engagement, and cognitive engagement, with a total of 32 options. Students were asked to report their learning engagement during their college English learning (e.g., I can prepare the English learning before class). The scores were rated using Likert 5-point scoring, from “1” strongly disagree to “5” strongly agree, the higher the score, the higher the student’s engagement. The scale underwent rigorous psychometric validation, including exploratory and confirmatory factor analyses. All indices met established thresholds for good model fit: Reliability: Cronbach’s *α* = 0.970, Sampling adequacy: KMO = 0.952, Sphericity: Bartlett’s test = 27815.222 (*p* < 0.05). Structural validity: *χ^2^/df* = 2.625, GFI = 0.932, CFI = 0.964, TLI = 0.941, IFI = 0.964, RMSEA = 0.030.

#### 3.2.4 ESE scale

The ESE scale developed by Chen (2018) includes four aspects: English learning ability efficacy, English learning effort efficacy, English learning challenge efficacy, and English learning setback efficacy, with a total of 22 options. Students were asked to report their self-efficacy during their college English learning (e.g., I persist in studying English for a certain amount of time every day). This scale adopts a Likert 5-point scoring system, ranging from “1” strongly disagree to “5” strongly agree, with higher scores indicating higher student’s self-efficacy. The scale underwent rigorous psychometric validation, including exploratory and confirmatory factor analyses. All indices met established thresholds for good model fit: Reliability: Cronbach’s α = 0.934, Sampling adequacy: KMO = 0.924, Sphericity: Bartlett’s test = 9813.776 (p < 0.05). Structural validity: *χ^2^/df* = 2.972, GFI = 0.926, CFI = 0.947, TLI = 0.932, IFI = 0.947, RMSEA = 0.039.

#### 3.2.5 ELS scale

Students’ English learning strategy was assessed with Oxford’s (1990) “Strategy Inventory for Language Learning Scale” (Chinese Version), which consists of 50 items and six dimensions: memory, cognition, compensation, metacognition, emotion, and communication. Students were asked to report their English learning strategy during the college English learning (e.g., In order to better memorize the words, I write down the new words on the card). The scores were rated using Likert 5-point scoring, from “1″ strongly disagree to “5″ strongly agree, with higher scores indicating more learning strategies. The scale underwent rigorous psychometric validation, including exploratory and confirmatory factor analyses. All indices met established thresholds for good model fit: Reliability: Cronbach’s *α* = 0.975, Sampling adequacy: KMO = 0.967, Sphericity: Bartlett’s test = 20430.614 (*p* < 0.05). Structural validity: *χ^2^/df* = 2.915, GFI = 0.928, CFI = 0.961, TLI = 0.952, IFI = 0.961, RMSEA = 0.041.

#### 3.2.6 ELP

Students’ ELP was from their reported scores of CET-4. CET-4 score is usually considered a qualification for measuring the English proficiency of non-English majors in China. It is one of the conditions for students to obtain a degree certificate upon graduation and also one of the thresholds that many employers require them to pass when looking for a job. It tests students’ comprehensive abilities in listening, speaking, reading, writing, and translation, with a total score of 710 points and a minimum score of 425 points for passing the exam. The average score (M ± SD) of CET-4 is 467.57 ± 43.32.

### 3.3 Data collection and analysis procedures

This study followed a rigorous empirical research protocol for data collection and analysis. During the data collection phase, the research team gathered measurement data on core variables (TCB, ELM, ELE, ESE, ELS) using standardized questionnaires. All scales were administered in the form of a 5-point Likert scale. Additionally, demographic information including participants’ gender, age, and major, as well as self-reported CET-4 scores, were collected. Students gave their informed consent before completing the questionnaire, which assured them of data confidentiality and its exclusive use for scientific research. The analytical approach progressed through three systematic phases. First, prior to analysis, we confirmed data missingness was completely random (Little’s MCAR test: *p* > 0.05) and implemented expectation–maximization imputation for minimal missing items (<5%). All variables met normality assumptions(skewness/kurtosis < |2.0|), with visual inspection of Q-Q plots revealing no substantial deviations. Second, descriptive analyses established scale reliability (Cronbach’s α range = 0.924 ~ 0.975) and bivariate correlations. Measurement invariance testing across demographic subgroups preceded primary inferential analyses. Third, Hypothesis testing employed Hayes (2012) PROCESS macro with maximum likelihood robust estimation to examine two theoretically-derived serial mediation pathways: (1) the motivation-engagement pathway (TCB → ELM→ELE → ELP) grounded in SDT and (2) the efficacy-strategy pathway (TCB → ESE → ELS → ELP) from SCT. All models incorporated 5,000 bootstrap samples for bias-corrected 95% confidence intervals while controlling for gender, major, and grade level.

## 4 Results

### 4.1 Common method deviation test

We conducted Harman’s single-factor test to assess common method bias across all measures (TCB, ELM, ELE, ESE, ELS). The exploratory factor analysis yielded 24 factors with eigenvalues greater than 1. The first factor accounted for only 21.774% of the total variance after rotation, which is substantially below the 40% critical threshold (Tang and Wen, 2020). These results suggest that common method bias does not pose a significant threat to our findings.

### 4.2 Descriptive statistics

After controlling for demographic covariates (gender, major and grade level), partial correlation coefficients revealed statistically significant positive associations among all variables (Table 2): TCB, ELE, ELM, ELS, and ELP (r = 0.10 ~ 0.71, *p* < 0.01). Specifically, TCB showed a significant positive correlation with CET-4 scores (r = 0.12, *p* < 0.01), thus supporting Hypothesis 1.

**Table 2:** Correlation analysis between variables.

Variable	Skewness	Kurtosis	M	SD	1	2	3	4	5	6
TCB	−0.841	0.752	4.33	0.53	1					
ELM	−0.134	−0.609	3.61	0.59	0.22^**^	1				
ELE	0.285	0.540	3.78	0.79	0.45^**^	0.55^**^	1			
ESE	−0.307	0.443	3.89	0.62	0.30^**^	0.48^**^	0.71^**^	1		
ELS	−0.118	−0.392	3.34	0.66	0.11^*^	0.10^*^	0.13^**^	0.12^**^	1	
ELP	−0.453	0.564	467.57	43.32	0.12^**^	0.15^**^	0.16^**^	0.15^**^	0.21^**^	1

*p < 0.05, **p < 0.01, ***p < 0.001.

### 4.3 Serial mediation analysis

For the mediation analysis, we employed Hayes’ PROCESS macro in SPSS 23.0, specifically utilizing Model 82 to examine the serial mediation effects. The bootstrap method with 5,000 resamples was applied to generate bias-corrected 95% confidence intervals for all parameter estimates. The serial multiple mediation analysis confirmed two motivational-cognitive chains. As presented in Table 3, the regression analysis yielded:(1) Equation 1 demonstrated that TCB exerted a significant positive effect on ELM (*β* = 0.222, t = 5.022, *p* < 0.001); (2) Equation 2 revealed significant positive effects of TCB on ELE (*β* = 0.346, t = 9.652, *p* < 0.001) and of ELM on ELE (*β* = 0.474, t = 13.365, *p* < 0.001); (3) Equation 3 indicated that TCB significantly positively predicted ESE (*β* = 0.307, t = 7.081, *p* < 0.001); (4) Equation 4 showed significant positive effects of TCB on ELS (*β* = 0.079, t = 2.633, *p* < 0.05) and of ESE on ELS (*β* = 0.089, t = 2.942, *p* < 0.01); (5) Equation 5 demonstrated significant positive effects of TCB (*β* = 0.052, t = 2.031, *p* < 0.05), ELM (*β* = 0.085, t = 2.899, *p* < 0.05), ESE (*β* = 0.056, t = 2.397, *p* < 0.05), and ELS (*β* = 0.206, t = 4.551, *p* < 0.001) on ELP, while the effect of ELE on ELP was non-significant (*β* = 0.026, t = 0.378, *p* > 0.05). These results provide empirical support for hypotheses H2, H3, H4, and H5.

**Table 3:** The serial mediation model of TCB and ELP.

Variable	Equation 1: ELM		Equation 2: ELE		Equation 3: ESE		Equation 4: ELS		Equation 5: ELP	
	β	SE	β	SE	β	SE	β	SE	β	SE
Constant	−1.526	0.843	−0.993	0.669	−1.810	0.826	−1.269	0.873	0.107	0.878
TCB	0.222^***^	0.044	0.346^***^	0.035	0.307^***^	0.043	0.079^*^	0.030	0.052^*^	0.026
ELM			0.474^***^	0.036					0.085^*^	0.029
ELE									0.026	0.071
ESE							0.089^**^	0.031	0.056^*^	0.023
ELS									0.206^***^	0.045
Gender	−0.104	0.100	0.141	0.079	0.091	0.098	0.043	0.013	0.006	0.004
Major	−0.217^*^	0.095	−0.142	0.075	−0.113	0.093	0.157	0.098	0.022	0.019
Grade	−0.023	0.078	−0.010	0.061	−0.003	0.076	−0.034	0.080	0.018	0.030
Age	0.101^*^	0.049	0.051	0.039	0.093	0.048	0.052	0.051	0.006	0.004
R^2^	0.071		0.419		0.106		0.026		0.101	
F	7.650^***^		59.661^***^		11.843^***^		2.187^**^		6.179^**^	

*p < 0.05, **p < 0.01, ***p < 0.001.

**Figure 2:**
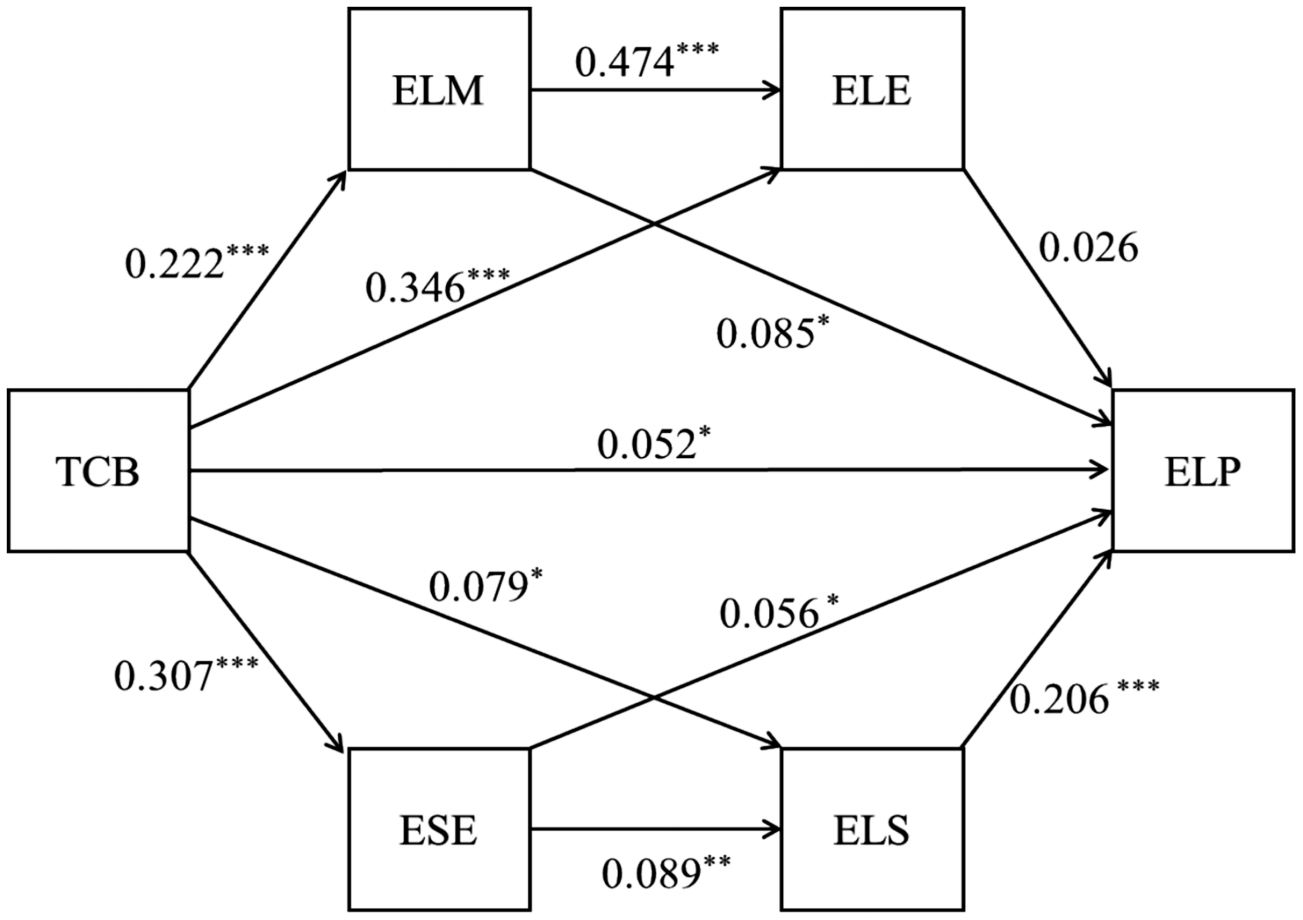
The serial multiple mediation model about the relationship between TCB and ELP through two sequential mediators. *p < 0.05, **p < 0.01, ***p < 0.001.

According to Table 4 and Figure 2, it can be seen that ELM, ELE, ESE, and ELS played a mediating role between TCB and ELP, with a total indirect effect value of 0.070, accounting for 57.38% of the total effect (0.122). The mediating effect consisted of six pathways: (1) ELM as a mediator, the mediating effect generated by TCB → ELM→ELP was 0.019 (0.005, 0.047) and the effect size was 15.58%; (2) The mediating effect of TCB → ELE → ELP, mediated by ELE was 0.009 (0.004, 0.061) and the effect size was 7.38%; (3) ESE as a mediator, the mediating effect of TCB → ESE → ELP was 0.017 (0.010, 0.058) and the effect size was 13.93%; (4) The mediating effect of ELS on TCB → ELS → ELP is 0.016 (0.009, 0.041) and the effect size was 13.11%; (5) The mediating effect of TCB → ELM→ELE → ELP, mediated by ELM and ELE, was 0.003 (0.001, 0.013) and the effect size was 4.92%; (6) The mediating effect of ESE and ELS as mediators was 0.006 (0.002, 0.019) and the effect size was 4.92%, which confirmed the hypotheses H6 and H7. At the same time, the Bootstrap 95% CI of the mediating effect did not include 0 and reached a significant level.

**Table 4:** The serial multiple mediating effect test of TCB and ELP.

Effect	Pathways	EV	ES	95%CI	95%CI
				Lower	Upper
Direct	TCB → ELP	0.052	42.62%	0.046	0.150
Indirect	TCB → ELM→ELP	0.019	15.58	0.005	0.047
	TCB → ELE → ELP	0.009	7.38	0.004	0.061
	TCB → ESE → ELP	0.017	13.93	0.010	0.058
	TCB → ELS → ELP	0.016	13.11	0.009	0.041
	TCB → ELM→ELE → ELP	0.003	2.46	0.001	0.013
	TCB → ESE → ELS → ELP	0.006	4.92	0.002	0.019
Total indirect		0.070	57.38	0.019	0.127
Total		0.122	100%	0.032	0.213

*p < 0.05, **p < 0.01, ***p < 0.001.

## 5 Discussion

### 5.1 TCB and ELP

This study found TCB positively predicted ELP (H1), which provides extra support for the existing research findings that TCB has a positive impact on academic performance (Lei et al., 2015), but extends his research in FL context. When teachers demonstrate caring behaviors during the teaching process, such as emotional attention to students, learning support, and daily concern, they can significantly stimulate students’ learning motivation, enhance their learning confidence, and reduce their negative emotional interference, such as learning anxiety (Wang, 2023) and academic pressure (Jiang et al., 2023). These positive emotional factors further promote students’ engagement and effort in English learning, thereby positively affect ELP. Therefore, TCB is an important factor in predicting ELP and teachers should pay more attention to the cultivation and implementation of caring behavior in teaching practice to improve students’ English learning effectiveness.

### 5.2 The mediating role of ELM in the relationship between TCB and ELP

Consistent with SDT (Deci and Ryan, 2000), TCB satisfies learners’ psychological need for competence and relatedness, thereby enhancing ELM (Opdenakker et al., 2012), which in turn fuels ELP (H2). This aligns with Ma et al. (2022) but extends their work by confirming ELM’s serial mediation with ELE (H6). This discovery reveals the intrinsic relationship between teacher care behavior, learning motivation, and English learning performance. TCB is conducive to creating a good teacher-student relationship. When teachers demonstrate care and support, it can affect students perceived teacher-student relationship, thus satisfying students’ psychological needs for relatedness and enhancing their feeling of competence, thus stimulating students’ learning motivation (Opdenakker et al., 2012; Ma et al., 2022). Learning motivation is an important driving force for students to engage in learning, overcome difficulties, and persist in studying. Students with higher learning motivation are more inclined to actively participate in learning activities, seek challenges, and continuously improve their English learning (Pajares et al., 2000; Liu et al., 2024). Therefore, learning motivation becomes a bridge between TCB and ELP, conveying the positive impact of TCB and promoting students’ performance and achievement in English learning. This discovery emphasizes the importance of TCB in stimulating and maintaining students’ learning motivation, as well as the critical role of learning motivation in improving students’ ELP.

### 5.3 The mediating role of ELE in the relationship between TCB and ELP

This study found that ELE played a mediating role between TCB and ELP (H3). It shows the intrinsic relationship between TCB, ELE and ELP. Building on Fredricks et al.’s (2004) engagement framework, TCB fosters emotional connections that elevate ELE (Perry et al., 2010; Wang, 2023), translating care into behavioral/cognitive engagement. This extends Sun’s (2021) findings by positioning ELE as a critical mediator in university EFL contexts. It establishes three key mechanisms through which TCB enhances ELP via ELE. First, emotional scaffolding. TCB creates a secure base for risk-taking in language use (Pishghadam et al., 2021). Teachers’ encouragement and emotional support can reduce their fear and anxiety in participating language tasks, thereby enhancing their intrinsic motivation and interest in learning (Perry et al., 2010). Two, behavioral activation. Supportive teacher behaviors enhance time-on-task by implementing structured accountability systems, such as progress tracking and peer monitoring, which strengthen students’ behavioral engagement through increased task commitment (Oga-Baldwin, 2019). Third, cognitive investment. Caring relationships promote metacognitive strategy use (Wang, 2023). Learning engagement is not only reflected in external behaviors, but more importantly, in students’ deep processing and positive thinking of learning content, thus actively employ different learning strategies. These changes in learning engagement further promotes students’ understanding and mastery of knowledge, improves learning efficiency and quality, and ultimately has a positive impact on academic performance (Skinner and Belmont, 1993; Lin and Wang, 2024). The discovery emphasizes the importance of teacher care behavior in enhancing students’ learning engagement, as well as the crucial role of learning engagement in improving students’ overall academic performance.

### 5.4 The mediating role of ESE in the relationship between TCB and ELP

Supporting Bandura’s (1977) self-efficacy theory, TCB bolsters ESE through mastery experiences (Zimmerman, 2000), verbal persuasion and emotional arousal regulation (Wang, 2023; Ding, 2024), confirming Lei et al.’s (2015) mediation model in FL settings (H4). Crucially, our study reveals ESE’s sequential linkage with ELS (H7)—a novel contribution. With teacher’s support, care, encouragement and inclusiveness, students can feel the ability in completing tasks, participating in class and achieving learning goals, thus enhancing their self-efficacy, which is a key factor affecting students’ learning motivation and academic performance (Schunk and DiBenedetto, 2021). Students with higher self-efficacy are more inclined to actively face learning challenges, persist in hard work, and exhibit better academic performance (Wang and Wang, 2025). Lei et al. (2015) found that there is a positive correlation between TCB and academic performance, with self-efficacy playing an important mediating role in it. Therefore, self-efficacy serves as a bridge between TCB and ELP, conveying the positive impact of TCB and promoting students’ performance and achievement in English learning. This discovery emphasizes the importance of TCB in enhancing students’ self-efficacy in the learning environment, as well as the crucial role of self-efficacy in improving students’ English learning outcomes.

### 5.5 The mediating role of ELS in the relationship between TCB and ELP

The results demonstrate that ELS significantly mediate the relationship between TCB and ELP (H5). This finding aligns with Oxford (1990) foundational work on strategy instruction in language learning but extends it by revealing the novel aspect that TCB’s multidimensional nature differentially activates distinct strategy types through behavior-specific pathways: For conscientiousness, teacher’s rigorous lesson planning, timely feedback etc. can activate students’ cognitive strategies, such as memorization techniques; For support, teacher’s individualized scaffolding can activate metacognitive strategies, such as goal-setting; For Inclusiveness, teacher’s validating diverse perspectives can activate affective strategies, such as anxiety/stress regulation (Wang, 2023). Unlike prior studies focusing on anxiety and stress reduction (Wang, 2023; Jiang et al., 2023), we demonstrate TCB’s proactive role in strategy cultivation. Specifically, cognitive strategies were enhanced through structured practice opportunities created by teachers’ task commitment; Metacognitive development emerged from scaffolded reflection during supportive interactions; Affective regulation was facilitated by emotionally safe environments fostered through inclusive behaviors.

Strategy use is very crucial for improving efficiency and achievement in FL learning (Teng and Yang, 2023; Cai et al., 2025). Students with more effective learning strategies can better organize and manage learning materials, solve problems more flexibly, and demonstrate higher autonomy in the learning process (Pintrich, 2000; Li et al., 2025). Therefore, learning strategies become a bridge between TCB and academic performance, conveying the positive impact of TCB and promoting students’ performance in learning. This discovery emphasizes the importance of TCB in guiding students’ development and effective learning strategies in language learning process, as well as the critical role of learning strategies in improving students’ overall academic performance.

### 5.6 The sequential mediation of ELM and ELE in the relationship between TCB and ELP

Building on SDT (Ryan and Deci, 2017), our findings establish a clear motivational pathway through which TCB enhances ELP. The sequential mediation model (H6), reveals how caring pedagogical practices initiate a cascade of positive learning processes, which helps us understand the importance of motivation-engagement relationship in language learning. TCB can create a good teacher-student relationship and a favorable learning environment for students. Good teacher-student relationships have been proven to stimulate students’ learning motivation (such as enhancing their interest, confidence, and goal orientation) (Ma et al., 2022). Learning motivation, as an internal driving force for students to engage in learning activities, has a significant impact on their learning attitude, effort, and academic achievement (Ding, 2024). When students feel the care and support from teachers, they are more likely to develop positive intrinsic motivation and initiate their emotional engagement in English learning. Meanwhile, learning engagement also plays an important mediating role between TCB and ELP. Learning engagement involves the time, energy, and focus that students invest in learning activities, and is an important indicator of their level of learning effort (Feng and Hong, 2022). When students feel the care, support and respect demonstrated by teachers, it can encourage students to increase their learning engagement, such as actively participating in classroom discussions, taking the initiative to complete assignments and review tasks beforehand, which in turn are helpful to improve students’ English learning performance. This discovery emphasizes the importance of teacher care behavior in stimulating students’ learning motivation and engagement, as well as the critical role of learning motivation and learning engagement in improving students’ overall academic performance.

### 5.7 The sequential mediation of ESE and ELS in the relationship between TCB and ELP

The cognitive-efficacy chain confirmed SCT (Bandura, 1977) and extends it by supporting that self-beliefs enable strategic action (Teng and Yang, 2023) (H7). This addresses Ma et al.’s (2017) call to explore serial mediations in FL achievement. This serial mediation pattern offers important insights into the psychological mechanisms through which TCB ultimately enhance language learning outcomes.

The current study advances theoretical understanding in three ways. First, we identify TCB can foster self-efficacy. Supportive feedback can help build students’ confidence in their language abilities, while inclusive classroom practices help develop resilience against setbacks. These suggest that teachers can strategically employ specific care dimensions to target different efficacy sources. Second, self-efficacy can translate into strategic behavior. Efficacy beliefs enable learners to select more appropriate strategies for given tasks, persist in strategy use despite difficulties, overcome negative feelings (Wang, 2023) and adapt strategies flexibly to changing demands (Cui and Hu, 2022). The use of these strategies further promotes the improvement of students’ English learning performance (Teng and Yang, 2023; Liu and Chen, 2024). This explains why efficacious learners typically outperform their peers. Third, it addresses Ma et al.'s (2017) call for investigating serial mediation in FL achievement by demonstrating how teacher behaviors initiate a chain reaction of psychological processes. Importantly, this pathway appears particularly strong for learners who initially doubt their language abilities, suggesting that teacher care may serve as a corrective for negative self-perceptions (Wang, 2023). Therefore, self-efficacy and learning strategies serve as a chain mediator between TCB and academic performance, conveying the positive impact of TCB and jointly promoting students’ positive performance in language learning. This discovery emphasizes the importance of TCB in enhancing students’ self-efficacy and utilizing effective learning strategies, as well as the crucial role of these two mediating variables in improving students’ overall academic performance.

In short, Unlike Ma et al. (2017) who found self-efficacy and strategies mediated good teacher-student relationship’ effect in middle schools, this study reveals sequential mediation (ELM→ELE; ESE → ELS) among university learners. This suggests TCB’s mechanisms evolve with learners’ maturity. Additionally, while Wang (2023) linked TCB to reduced anxiety, our model demonstrates its proactive role in fostering motivational-cognitive pathways to ELP.

## 6 Implications

This study showed that teacher care behavior had a significant positive prediction on students’ English learning performance. This predictive effect is achieved through multiple mediating pathways of learning motivation and learning engagement, as well as self-efficacy and learning strategies. This discovery not only reveals the important role of teacher care behavior in students’ FL learning, but also provides theoretical and practical basis for FL educational practice. Theoretically, the validated serial mediation model extends SDT and SCT by integrating them into a unified framework for FL contexts, highlighting TCB’s role in activating sequential psychological processes. Practically, teachers should embed care-driven practices (e.g., personalized feedback, emotional support) into pedagogy. Institutions must train educators to cultivate students’ self-efficacy via strategy instruction (e.g., metacognitive workshops) and foster engagement through collaborative tasks.

## 7 Limitations and future directions

Although this study has yielded significant findings, it has several limitations that suggest important directions for future research: First, sample characteristics. The overrepresentation of female students and humanities majors from Chinese universities limits generalizability to other populations. Future studies should include more balanced samples, particularly from STEM fields and vocational education contexts where language learning needs may differ; Second, research design. Our cross-sectional design cannot establish causality. Longitudinal studies tracking students across academic years would better reveal how teacher care behavior influences developmental trajectories of motivation and self-efficacy; Third, measurement approaches. While using validated scales, reliance on self-reported data (especially CET-4 scores) may introduce bias. Multimethod approaches incorporating classroom observations, teacher reports, and standardized tests would provide more robust evidence. Fourth, contextual factors. Findings reflect China’s exam-oriented EFL context. Cross-cultural comparisons with non-exam environments (e.g., immersion programs) could clarify how educational systems moderate TCB’s effects.

To address these limitations and extend our findings, future research should prioritize the following directions: First, testing the model in diverse institutional contexts (K-12 to workplace training); Second, developing mixed-methods designs combining longitudinal surveys with classroom ethnography; Third, investigating cultural moderators through international collaborations.

## 8 Conclusion

This study delves into the complex relationship between teacher care behavior, learning motivation, learning engagement, self-efficacy, learning strategies, and learning performance. Through empirical analysis and literature review, we found that teacher care behavior not only directly had a positive impact on students’ academic performance, but also indirectly promotes students’ academic achievement through multiple mediating mechanisms. Specifically, this care behavior can stimulate students’ learning motivation, thereby enhancing them to invest more time and energy in the learning process, and focus more on their studies; At the same time, the care and support of teachers can enhance students’ self-efficacy, encourage them to face learning challenges more actively, guide them to develop and apply more effective learning strategies, and improve learning efficiency and grades. In summary, this study indicates that teacher care behavior plays a crucial role in students’ learning and growth processes, influencing their academic achievement and overall development through various means. Therefore, in educational practice, teachers should focus on demonstrating caring behavior, providing emotional support and encouragement to students to promote their learning and growth.

## Data Availability

The original contributions presented in the study are included in the article/supplementary material, further inquiries can be directed to the corresponding author.
